# “This Is My Future?”: Understanding the Lives of Emerging Adult Women Living with Chronic Pain Through a Narrative Inquiry

**DOI:** 10.1177/23333936251335531

**Published:** 2025-04-17

**Authors:** Jenise Finlay, Aniela M. dela Cruz

**Affiliations:** 1 University of Calgary, AB, Canada

**Keywords:** chronic pain, emerging adult women, identity, narrative inquiry, nursing, qualitative, young adult women, Canada

## Abstract

Chronic pain disproportionately affects women yet is often underestimated by medical professionals. In Canada, chronic pain rates have risen significantly, particularly among those aged 20 to 29 without other health conditions. However, limited qualitative research focuses on chronic pain exclusively in women under 30. By focusing on gender, this narrative inquiry study examined how societal narratives and stereotypes uniquely affect emerging adult women’s experiences of chronic pain, contributing to their dismissal and invisibility in both personal and institutional contexts. Two key narrative threads were co-created with participants through analysis of their stories: silenced, invisible, and locating self with pain, and resisting singular stories of people living with chronic pain. Participants’ shared family narratives of dismissal, stories of being silenced in health care, and dominant narratives in the context of age and gender that shaped the participants’ stories to live by. This study demonstrates the importance of recognizing people in the midst of living with chronic pain. Understanding unique pain experiences during emerging adulthood can improve treatment options and long-term outcomes for this demographic.

## Introduction

Approximately one in five Canadians live with chronic pain, yet it remains a stigmatized and invalidated condition despite being the third-largest health problem worldwide (Canadian Pain Task Force [CPTF], 2019; Jackson et al., 2014). *Chronic pain* has been defined in the literature as pain persisting or recurring beyond either 3 or 6 months in minimum duration (Raja et al., 2020). Chronic pain interferes with one’s ability to function and participate in workplaces, relationships, schools, and society (Institute of Medicine, 2011; Racine et al., 2014). In 2019, one-third of Canadians with chronic pain surveyed knew someone who had taken their own life because of uncontrolled pain (Chronic Pain Association of Canada, 2019). By focusing on gender, this narrative inquiry study examined how societal narratives and stereotypes uniquely affect emerging adult women’s experiences of chronic pain, contributing to their dismissal and invisibility in both personal and institutional contexts.

Chronic pain in emerging adults is a common problem, affecting up to 30% of those aged 18 to 39 (Brown et al., 2021; Mills et al., 2019). *Emerging adulthood* is the development period between adolescence and adulthood, typically spanning ages 18 to 29 (Arnett, 2007). During this time, the importance of peer relationships and social context are heightened, and individuals living with chronic pain may miss opportunities and experience negative peer interactions, leading to decreased psychological well-being (Arnett, 2007; Twiddy et al., 2017).

Women of all ages with chronic pain report a greater number of health problems, complex symptoms, mental health and/or long-term symptoms than men (Ballweg et al., 2010; Briggs et al., 2022; Werner et al., 2003). Both patient and health provider characteristics influence existing gender bias in the health care system (Bartley & Fillingim, 2013), and conditions where pain is the only reported symptom predominantly affect women (Samulowitz et al., 2018). These “medically unexplained” conditions are associated with disbelief of women’s pain (Samulowitz et al., 2018, p. 5). Consequently, women are more likely to be dismissed and labeled as “emotional, psychogenic, hysterical, or oversensitive” (Ballweg et al., 2010, p. 4). Emerging adult women in particular are at higher risk in primary care for insufficient pain management (Green & Hart-Johnson, 2010), and face unique challenges navigating chronic illness, dating, body image, college, careers, establishing independence from family, and bearing children at an age where youth is equated to being healthy (Hirsch, 2018; Stinson et al., 2013). Nevertheless, the majority of adult chronic pain research focuses on individuals middle-aged and older (Brown et al., 2021).

Chronic pain disproportionately affects women, and their pain is often dismissed or minimized due to gendered stereotypes that frame women’s pain as emotional or exaggerated rather than physical (Bendelow, 1993; Denny, 2009). Research has shown that women frequently struggle to have their pain legitimized, navigating cultural narratives that treat physical pain as “normal” or inevitable for women, particularly during menstruation or reproductive years (Bendelow, 1993; Denny, 2009). Ahlsen et al. (2014) further highlight how these gendered narratives influence the ways women construct their identities in the context of chronic pain, often resisting societal expectations of femininity that conflate endurance with strength. In this study, *gender* was defined as the socially constructed roles, behaviors, and expectations associated with femininity, masculinity, and other identities. These constructions influence how chronic pain is perceived, validated, and managed within healthcare systems.

While much of the existing literature focuses on middle-aged or older women, the experiences of emerging adult women navigating chronic pain remain underexplored. This study addresses this gap by examining how gendered and age-related narratives shape the pain experiences and identities of emerging adult women living with chronic pain in Canada, utilizing Clandinin and Connelly’s (2000) narrative inquiry methodology.

Central to Clandinin and Connelly’s (2000) narrative inquiry is the idea that human experience is shaped through stories. *Being storied* refers to how individuals are perceived and positioned within broader societal, familial, and institutional narratives that shape their identity and lived experiences. These narratives are not static but evolve over time as individuals negotiate their identities within relational and social contexts. Similarly, *stories to live by* captures how individuals make sense of their experiences through personal narratives that intersect with social and cultural narratives. These concepts provide a framework for understanding how the participants in this study navigated the interplay between chronic pain, identity, and societal expectations.

## Research Design

In this article, key research findings are discussed from a Master of Nursing thesis research study which received ethics approval from the University of Calgary Conjoint Health Ethics Board (REB21-0540) on June 2, 2021. All participants provided written, informed consent prior to enrollment in the study.

### Methodological Approach

Clandinin and Connelly’s (2000) narrative inquiry is a qualitative, relational methodology that aims to understand identity by examining how people make sense of their life through storytelling. This approach is particularly poignant in understanding the nuanced experiences of emerging adult women living with chronic pain—a deeply personal and often misunderstood condition. Central to narrative inquiry is the premise that human experience itself is a primary source of knowledge.

Unlike other narrative methods that focus more on structural or thematic elements of stories (Holloway & Freshwater, 2007), Clandinin and Connelly’s (2000) narrative inquiry is uniquely suited for our study due to our focus on the temporal, social, and situational dimensions of experience. This approach is imperative in chronic pain research, where personal and contextual stories significantly influence health outcomes and identity perceptions (Barnes, 2018; Smith & Sparkes, 2009). In addition, this form of narrative inquiry positions the researcher and participant as integral to the research landscape. This methodology foregrounds key ethical considerations such as reflexivity, reciprocity, and mutual vulnerability, ensuring a depth of engagement between both the researcher and participant’s lived realities (Caine et al., 2022; Clandinin et al., 2018).

In narrative inquiry, the researcher situates themselves alongside participants throughout the research process, engaging relationally to co-construct knowledge. This involves attending to the relational, temporal, and social contexts that shape participants’ experiences, as well as reflexively examining how the researcher’s own positionality influences the research. In this study, the researcher maintained ongoing dialogue with participants, revisiting and revising their narratives collaboratively to ensure their voices were accurately represented. This relational approach emphasizes the importance of mutual trust and respect in uncovering nuanced understandings of participants’ lived experiences. In this study, the focus was on how participant experiences living with chronic pain have been, and are, shaped by time, social context, and place, and how the participant positions themselves in these dimensions in their retold stories. This alignment with Clandinin and Connelly’s (2000) approach enabled an exploration of the intersection between chronic pain and identity formation in emerging adult women, highlighting how their stories evolve and intersect within the dimensions of time, place, and context.

### Methods

The concepts of *being storied* and *stories to live by* informed both the study design and data analysis (Clandinin, 2013). Being storied guided the exploration of how participants’ experiences were shaped by societal and institutional narratives that framed chronic pain as less credible for young women. Stories to live by provided a lens for understanding how participants reinterpreted and reclaimed their narratives in response to dismissal and invisibility. Data analysis focused on identifying narrative threads that connected participants’ personal stories to broader cultural, familial, and healthcare contexts.

#### Sampling, Recruitment, and Participants

Purposive sampling was used to recruit participants. Recruitment was conducted through the University of Calgary’s research recruitment website and three rehabilitation and wellness clinics. Inclusion criteria were: (a) aged 18 to 29, (b) female sex and identifying as a woman, (c) self-report living with persistent non-malignant pain for greater than 3 months duration at the time of recruitment, (d) oral English proficiency, (e) has sought health care related to the pain, and (f) able to provide informed consent for study participation. In narrative inquiry study, a small sample size is appropriate for multiple, in-depth conversations with each participant (Connelly & Clandinin, 2006; Kim, 2015). Between June and December 2021, 37 women expressed interest in the study. Due to the scope and timeline of this Master’s thesis, the first three participants who met all inclusion criteria and provided informed consent were selected. Unfortunately, one participant was lost to follow-up, leaving two participants—Hailey^1^ and Megan^1^—who fully engaged in the study. This approach and sample size aligns with narrative inquiry methodology, which prioritizes in-depth engagement with participants over breadth and generalizability.

#### Data Generation

Clandinin and Connelly (2000) describe data collection as *data generation* and can include conversations, field notes, written personal narratives, the researchers’ field notes and reflective journals, as well as participant artwork and other documents. In this study, participants individually met with the first author for four in-depth conversations, held virtually via a secure university-licensed Zoom account due to public health measures during the COVID-19 pandemic. Field notes, emails, researcher-created annals, and participant-created artifacts (e.g., artwork, essays, pain journals, and poetry) were also used as data. One participant provided her clinic records and consented to the use of these as data.

#### Data Analysis

Data analysis was done according to methods described in Clandinin (2013). Conversation transcripts were read and reread successively. Narrative accounts were developed in written form, based on each participant’s transcripts, field notes, photographs, and/or journals. These narrative accounts were formed in negotiation with participants to identify and interpret shared experiences, paying close attention to how the narratives were situated in time, sociality, and place (Clandinin & Caine, 2013). After developing each participant’s narrative account, the context of time, sociality, and place was used to identify narrative threads (Connelly & Clandinin, 2006). Narrative threads are central narratives or patterns co-constructed with participants that reverberate across individual narrative accounts; these threads connect stories and experiences to broader social, cultural, familial, linguistic, and institutional narratives, shaped by the researcher’s onto-epistemological and theoretical approaches (Clandinin, 2013). To ensure rigor, participant feedback was continuously sought to confirm accuracy and meaning throughout the research process, while guidance from relational response communities provided critical feedback to refine the research and its interpretation.

## Findings

Across participant narrative accounts were shared experiences of becoming emerging adult women living with chronic pain. In this paper, we focus on two of the three narrative threads that resonated across the narrative accounts of the participants, Hailey and Megan: (a) silenced, invisible, and locating self with pain, and (b) resisting the singular stories of people living with chronic pain. Hailey was 18 at the time of data generation and has lived with chronic pain since about age 10, suffering from fibromyalgia, migraines, irritable bowel syndrome, frequent urinary tract infections, and symptoms of interstitial cystitis. Megan was 25 at the time of data generation and has lived with chronic pain since age 12, having been diagnosed with idiopathic scoliosis, adenomyosis, and endometriosis.

### Silenced, Invisible, and Locating Self with Pain

Resonant narratives between participant accounts share dismissal experienced over time through relationships with family, friends, and health care providers. Hailey and Megan both experienced dismissal of their pain as a lack of validation, autonomy, belief, empathy, and equality. Field notes recorded during interviews documented moments of emotional expression, such as Hailey pausing as she described the toll of chronic pain on her relationships. These observations provided deeper context to her narrative and highlighted the emotional weight of her story, which was further explored in subsequent conversations.

For Hailey, dismissal of her pain began as a child; she told stories of “tough love” and a lack of concern for her pain that evolved into self-doubt and experiences of being silenced in health care. Megan’s narratives of dismissal first formed during her time as a child at the scoliosis clinic, a place that molded her early narratives of living with chronic pain. These experiences of not having their pain recognized or understood evolved through Hailey and Megan’s social, cultural, personal, and broader institutional narratives storied in spaces of family and health care.

#### Family Narratives of Dismissal

While research specifically focusing on emerging adults is limited, studies on children and adolescents with chronic pain suggest that experiences of misunderstanding and disbelief from family members are common and can lead to disruptions in family relationships. Familial narratives shared by Hailey and Megan centered primarily around interactions with their mothers, who were their caregivers in their chronic pain journeys until adulthood. For Hailey, when she was officially diagnosed with fibromyalgia, her mother did not accept the diagnosis. In her narratives, Hailey made sense of these tensions as her mother wanting to do everything she could to find an alternate diagnosis for Hailey. She explained that her mother dismissed fibromyalgia as “not a real disorder” out of love and a desire to protect Hailey from living with a stigmatized condition.

Similarly, Megan’s mother dismissed her acceptance of disability. Megan explained,

[. . .] my mom was very like, “you’re not disabled”, because in her mind again, like in her mind this was to protect me because, if I was disabled, then, you know, people would look down on me forever and stuff but, and so it took me a while to accept, you know what? I am a disabled person and that’s okay.

Megan interpreted this as her mother’s protective instincts and an attempt to shield her from societal judgment, which Megan explained as the belief that people would “look down on her forever” if she identified as disabled. Despite these intentions, this led to tensions between how Megan storied herself and how her mother storied disabled people.

Megan also described how her father’s experience with pain shaped her own. She explained that her father’s pain was often perceived as more severe, which influenced her to downplay her own pain. “I always compared my pain to his, thinking mine was less valid,” she shared. These intergenerational narratives, coupled with her father’s experiences in healthcare, contributed to Megan’s effort to portray herself as a patient who avoided being dismissed or labeled as a drug-seeking individual.

Growing up, both Hailey and Megan were expected to continue chores and daily tasks regardless of their pain levels. Megan reflected on this, stating, “I feel like I’m expected not to complain.” This echoed familial and cultural narratives that women are expected to work through sickness or pain: “Women are expected to still do things like, you know, when you’re sick, you’re still supposed to manage your house and take care of your kids,” Megan explained. These dominant narratives have shaped Megan’s ongoing struggle to balance self-compassion with the cultural expectations of productivity while managing her pain.

While Hailey re-storied these dismissal experiences as “tough love,” showing gratitude for her parents’ intention to foster independence, both Hailey and Megan acknowledged the lasting impact of these narratives. Megan further recalled intergenerational expectations passed down from her grandmother to her mother: the belief in continuing to work despite pain or illness. Together, these familial and cultural narratives shaped their understanding of pain and disability, reinforcing the notion that disability was a deficit and that women were expected to persist in their responsibilities despite their pain.

#### Making Sense of Pain Through Family Relationships

In their narrative accounts, Megan and Hailey described how their relationships with their families shaped—and were shaped by—their pain experiences as children and teenagers. Megan recounted her mother’s anger and frustration at how a scoliosis physician dismissed Megan’s pain. Similarly, Hailey described her mother’s anger at the dismissal Hailey experienced during her first psychiatric consultation. These moments illustrate how the emotions and reactions of their mothers influenced Hailey and Megan’s experiences of pain as children and teenagers.

As Megan grew older, her narratives shifted to reflect her increasing independence. She shared how her mother’s role in her pain journey diminished as she transitioned to university and began advocating for herself. Despite this independence, Megan was expected to look after her younger sister at university, and these stories of responsibility created feelings of anxiety and stress, contributing to increased pain for Megan. “When I couldn’t help her because of my pain, I felt so guilty,” Megan explained, reflecting on the expectations she placed on herself and those she felt were set by her mother.

Hailey’s narrative account made visible the ways her parents did not acknowledge her pain experience. Her stories of pain as a child were met with a lack of empathy, and Hailey stopped mentioning her pain to her parents, establishing her understanding of constant pain as a normal human experience. “I thought I was normal until I started talking about it to my friends,” Hailey reflected on her pain. Over time, as Hailey grew into a teenager, her narratives shifted to experiencing pain dismissal from her parents for other reasons. Her parents attributed her chronic pain to her eating disorder, and they interpreted her limited ability to move as a lack of willingness to improve mentally. Additionally, her parents believed her migraines were solely an excuse not to attend school. Over time, these experiences contributed to Hailey understanding the stigma of fibromyalgia as “not a real disorder.” Physical touch became painful for Hailey in high school, creating tensions with her mother, who according to Hailey, did not understand her pain. When Hailey had to quit skiing because of her health, her father was dismayed. Disappointment from both her parents related to her mental and physical health influenced Hailey’s perception of herself, becoming a young person living with pain, and disappointing others.

#### Stories of Being Silenced in Health Care

Megan and Hailey’s experiences of dismissal extended beyond their families and into healthcare settings, where they encountered systemic invalidation of their pain. Megan described her early experiences at the scoliosis clinic, where she was repeatedly told that “scoliosis does not cause pain.” These interactions, which occurred during her teenage years, shaped her understanding of pain as something that was dismissed and not believed.

Email exchanges with Megan revealed additional reflections about her healthcare experiences. In one email, Megan elaborated on the frustration she felt when her concerns were dismissed by physicians, stating, “It feels like they just see me as young and dramatic.” This added nuance to her narrative of dismissal and invisibility. Over time, Megan began concealing her pain, reflecting the influence of these institutional narratives on her identity as a patient.

Hailey also shared stories of being silenced in healthcare, particularly as her pain was attributed to other diagnoses. Her pain was not only silenced by health providers, but she also became storied by health care providers as “not doing enough” or that her pain was a result of her eating disorder, depression, trauma experiences, and/or anxiety. These interactions contributed to Hailey’s identity of herself as a woman living with pain as “not a fully functional human,” ascribing her pain as her fault and affecting her self-esteem. “It’s like a checklist,” she explained, “like, ‘Are we going to take you seriously? One, she’s female; two, she’s queer; three, she’s on her period; four, she has other diagnoses,’” indicating how her gender, sexual orientation, and psychiatric diagnoses were used to invalidate her pain. These experiences led Hailey to feel as though her pain was her fault, a narrative that impacted her self-esteem and how she viewed herself as a woman living with chronic pain.

Both Megan and Hailey described the additional burden of appearing credible in healthcare settings and experienced such tensions and struggles through their early and ongoing experiences of living with pain. For Megan and Hailey, having a medically recognized diagnosis for their pain allowed them to make sense of who they were becoming as emerging adult women living with pain. As Hailey stated, having a diagnosis meant having a “word that describes all of it.” Having a medically acceptable cause for their pain meant it was no longer something purely psychological, thus considered “real” or legitimate, and this shaped acceptance of their pain as part of who they were becoming, encouraging them to move forward with their lives.

Experiencing dismissal for her eating disorder, fibromyalgia, gender, and sexual orientation, Hailey still finds it difficult to discuss her pain with care providers. Hailey stated, “pretty much all of my pain was dismissed by mental health and now all of my mental health is dismissed by my pain. Honestly, like, there, you cannot have both at the same time.” Megan and Hailey stated that they prefer female physicians because of past experiences with male physicians where they felt dismissed for their pain. Megan noted the challenges of standing up to physicians, reflecting on the cultural reverence given to doctors: “It’s hard to stand up to your doctor, especially for the first time,” she explained. Hailey echoed this, sharing how being assigned a male doctor in the emergency room would immediately create anxiety about whether her pain would be taken seriously. “If I’m in the ER and realize that I’m, I’ve been assigned a male doctor like. It’s just like please, please listen to me [. . .].” Hailey’s experiences have led her to assume each patient-provider relationship begins with her being seen as not credible or trustworthy. These narratives of dismissal in health care became Hailey’s stories to live by, influencing her understanding of herself as an emerging adult woman whose pain is something to be dismissed or silenced. Both Megan and Hailey stated they now avoid discussions about their pain unless initiated by the health care provider.

#### Self-Doubt and Dismissal: The Consequences of Being Storied by Others

Having experienced invalidation and dismissal of their pain over time in familial and healthcare settings, Hailey and Megan’s narrative accounts demonstrate how these experiences fostered self-doubt and internalized dismissal. Hailey contributed a piece of poetry reflecting her experience of living with chronic pain. The poem depicted her feelings of invisibility and frustration, with lines such as, “They see a young body but not the cracks beneath.” This artifact provided a vivid, creative expression of her narrative and added depth to the analysis of her identity formation. Hailey also shared how she began to doubt the validity of her pain, wondering if it was real, imagined, or psychogenic, as it had been storied by others. She reflected,

I started paying attention to it and it was like if somebody puts their hand on my arm it hurts, and I was also like, is that pain? Or not? Because I had like this false perception of what pain is apparently [. . .] but I also just ignored it ‘cause I was like I’m just being weird and stuff and I also had like a lot of mental health stuff that wasn’t even being picked up on [. . .]

These narratives of doubt, reinforced by repeated invalidation, shaped how she perceived herself and her pain.

Megan’s struggle with self-validation was similarly shaped by dismissal from others. She shared how she wished her pain was more severe and visible to others, because the invisibility of her condition led her to question its legitimacy. These struggles with self-compassion and validation affected both Megan and Hailey’s ability to care for themselves. Megan explained, “Sometimes I feel like I need permission to take pain medication or rest.” These experiences of invalidation led to an internalization of societal and familial narratives, making it challenging for both participants to prioritize their own needs without guilt.

The narrative accounts within this section collectively demonstrate the multifaceted challenges faced by Hailey and Megan in navigating chronic pain. From familial dismissal to systemic silencing in healthcare, these experiences shaped how the participants storied themselves and their pain. However, their narratives also illustrate resilience and agency, as both women worked to re-story their experiences and reclaim their identities as emerging adults living with chronic pain.

### Resisting the Singular Stories of People Living with Chronic Pain

Hailey and Megan’s narrative accounts illustrate how they live alongside dominant societal and institutional narratives about people living with chronic pain. These narratives often paint individuals with chronic pain as less competent, less worthy, or unable to fully participate in life, and purport that chronic pain only accompanies aging and visible disability. Megan and Hailey have storied themselves according to these dominant narratives, however, they have experienced tensions within their stories, in different spaces over time, as they sought to define themselves outside of these restrictive dominant narratives.

#### Challenging the Narrative of Disability as Incompetence

Megan and Hailey’s accounts highlight the dominant narrative that positions individuals with chronic pain as inherently less capable. Megan reflected,

I never really had the opportunity to know who I was without pain [. . .] it’s hard to know who am I, and who, who is my pain [. . .] It’s an identity almost for me like I didn’t lose anything because it wasn’t really like I was a person, but I wasn’t really a person before, like I was a kid right. So it’s more like I feel like I’d have more identity loss if I was no longer in pain.

She described how her identity had always been intertwined with her pain, but she resisted narratives that suggested this made her less capable or valuable. “It’s an identity almost for me,” Megan explained, adding that her pain had shaped her but did not define her entirely.

Hailey echoed this resistance, sharing how she navigated public and private spaces while confronting these narratives. She recounted how, on public transit, peers and strangers dismissed her pain because it was not visibly apparent. These experiences reinforced feelings of invisibility, but Hailey worked to challenge this narrative by asserting herself in relationships and social spaces. “Despite what people might think, I can still be in a relationship, and I can still achieve my goals,” she explained.

Both participants resisted dominant narratives that equate youth with health and chronic pain with incapacity. They actively re-storied their experiences to demonstrate their abilities and agency. Hailey shared her experience of participating in an intensive, multi-day canoe trip despite her pain, describing it as a way to prove to herself and others that she was capable. Similarly, Megan spoke of her achievements in academia and her personal relationships, stating, “Living with chronic pain hasn’t stopped me from pursuing what I want.”

#### Navigating Gendered Expectations

Hailey and Megan’s experiences of chronic pain were deeply influenced by gendered expectations. Megan described the pressure to minimize her pain: “I feel like I’m expected not to complain. Women are just supposed to carry on with everything, even when they’re in pain.” These familial expectations, passed down through generations, shaped her approach to managing pain. She elaborated, “My mom always had this attitude that you push through no matter what. I think it’s something she got from my grandma.”

Hailey also shared how cultural narratives diminished the credibility of her pain. “Pretty much all of my pain was dismissed by mental health, and now all of my mental health is dismissed by my pain,” she explained, illustrating how being both a woman and someone with overlapping chronic pain and mental health challenges compounded her struggles to be believed.

Despite these challenges, both participants resisted these narratives. Megan explained her efforts to balance self-care with societal expectations: “I’ve learned to give myself permission to rest, but it’s still hard not to feel like I’m failing when I take time for myself.” Hailey added, “I’m trying to unlearn the idea that I have to keep going, no matter what.” These insights reflect their journeys toward self-compassion and agency within a culture that often expects women to endure pain without complaint.

#### Redefining Intimacy and Relationships

For Hailey and Megan, chronic pain reshaped their experiences of intimacy and relationships, requiring them to navigate trust and communication carefully. Hailey reflected on the complexities of discussing her pain with partners: “If I am going to have a sexual relationship with someone, I need them to let me explain in detail, like what my pain is like, and I need to be able to trust them that they understand.”

Megan also shared her struggles with intimacy, particularly as it related to managing pain during sex. She described how pain and anxiety created a “never-ending feedback loop”: “You have pain, and then you’re anxious about having it again, and so it gets worse.” Megan emphasized the importance of mutual understanding in relationships, noting that her ability to maintain intimacy was grounded in communication and support from her partner.

Both participants challenged societal narratives that framed individuals with chronic pain as less capable of maintaining meaningful relationships. Hailey explained, “Intimacy is more than just the physical—it’s about feeling seen and understood.” Megan stated, “Living with chronic pain doesn’t mean settling for less in relationships. It’s about finding someone who respects and supports you.” These reflections highlight how Hailey and Megan redefined intimacy and resisted narratives that marginalized their relational experiences.

#### Stories to Live By

Hailey and Megan’s stories to live by demonstrate the interplay between societal narratives, familial expectations, and their evolving identities as emerging adult women living with chronic pain. Narrative inquiry emphasizes that stories to live by are shaped relationally, socially, and temporally, as individuals make sense of themselves within broader cultural and institutional narratives (Clandinin & Connelly, 2000). For Hailey and Megan, these stories were influenced by dominant narratives that framed chronic pain as less credible and dismissed their experiences as emerging adult women.

Megan described how her early experiences of dismissal in healthcare shaped her self-perception. “I feel like I’m always being told my pain isn’t real. It’s like I have to constantly prove myself, and it’s exhausting,” she shared. Over time, these experiences became part of her stories to live by, as she began to question her own credibility and worth in healthcare interactions. Megan reflected, “I started doubting myself because if no one else believed me, maybe I was making it up.”

Hailey echoed similar struggles, recounting how societal narratives about chronic pain as psychological influenced her identity. She explained, “It’s hard to feel like a whole person when everyone keeps telling you your pain is in your head.” These dismissals led Hailey to internalize feelings of self-doubt, but they also motivated her to reclaim her narrative. “I’ve learned to trust myself more. My pain is real, even if no one else thinks so,” she stated, reflecting a shift in her stories to live by.

For both participants, the process of re-storying their lives involved resisting narratives that framed their pain as illegitimate or their identities as diminished. Hailey and Megan shared how receiving a diagnosis helped them validate their experiences and begin to rewrite their stories. Hailey explained, “Having a diagnosis doesn’t fix everything, but it gives me something to point to. It’s a way of saying, ‘This is real.’” Similarly, Megan shared how her diagnosis allowed her to reframe her identity: “It doesn’t define me, but it’s part of who I am. I’m not broken; I’m just different.”

Hailey and Megan’s stories to live by also reflected their efforts to navigate societal expectations and assert their agency. Megan shared how she resisted the narrative that individuals with chronic pain cannot lead fulfilling lives: “I’ve learned to live with my pain, but it doesn’t stop me from being who I want to be.” Hailey echoed this sentiment, explaining, “Pain is part of my story, but it’s not the whole story. I can still have joy, relationships, and a future.”

## Discussion

Exploring disparities in how chronic pain is perceived and managed among emerging adult women illustrates the gender and age-related biases pervasive in healthcare. Guided by narrative inquiry methodology, this study positioned participant accounts as co-constructed interpretations shaped by relational, temporal, and social contexts. Building on work by Samulowitz et al. (2018) and Racine et al. (2014), our research highlights the nuanced experiences of women with chronic pain, showing that women are often stereotyped as emotional or hysterical and face significant challenges in being taken seriously by healthcare providers.

As Rice et al. (2024) and Bendelow (1993) established, women’s pain is frequently minimized due to societal and institutional biases that frame it as emotional or psychological rather than physical. These findings resonate with the narratives of Megan and Hailey, who described how their pain was often dismissed as exaggerated or imagined. Megan’s recounting of being told that “scoliosis doesn’t cause pain” and Hailey’s experience of having her pain misattributed to mental health reflect these systemic biases. These dismissals align with Denny’s (2009) work on the trivialization of women’s pain and the uncertainty many women experience in obtaining a legitimate diagnosis. By examining these stories within the framework of gendered narratives, this study builds on existing literature, illustrating how age-related dismissal intersects with cultural perceptions of femininity and pain.

Ahlsen et al. (2014) highlight how women’s narratives often resist gendered expectations by re-storying their experiences to assert agency. For Megan and Hailey, this resistance was evident in their efforts to reclaim their identities despite the pervasive cultural narrative that emerging adult women are “too young” to experience chronic pain. Megan’s frustration with being labeled as overreacting and Hailey’s struggles to advocate for herself reflect the enduring impact of these stereotypes. These experiences demonstrate the need for healthcare providers to listen to and validate the lived experiences of women with chronic pain.

Emerging adult women face unique challenges in navigating chronic pain because this life stage is often culturally equated with health, vitality, and independence (Hirsch, 2018). Participants in this study described how societal narratives of youth as a time of good health intensified the dismissal of their pain by peers, healthcare providers, and even family members. For example, Megan reflected on being repeatedly told that her pain “wasn’t real,” which reinforced feelings of invisibility. Similarly, Hailey described having her fibromyalgia symptoms dismissed as psychological, intensifying her isolation and making advocacy difficult. These findings align with existing research but highlight how dismissal during emerging adulthood can be particularly impactful, as this period is critical for identity formation, career development, and relational transitions (Barnes, 2018; Twiddy et al., 2017). Such experiences may not be as prominent for older women, whose chronic pain might be perceived as more legitimate due to cultural associations between aging and illness. This societal presumption contributes to a significant gap in recognizing and treating chronic pain in emerging adults, as they are often met with skepticism and suspicion, reinforcing fears of being labeled as malingering (Samulowitz et al., 2018).

Historically, women’s experiences of pain and psychological distress have been pathologized and dismissed, often leading to invasive medical interventions that sought to control or silence them (Connell, 2012; Levinson, 1976). Practices such as hysterectomies and lobotomies exemplify how women’s pain and emotions were frequently interpreted through a gendered lens, framing them as irrational or overly emotional. These historical patterns resonate in contemporary narratives of chronic pain experienced by women, where their symptoms are often minimized or attributed to psychological causes rather than recognized as valid medical conditions (Keogh, 2021; Walker et al., 2022).

This study contributes to ongoing conversations about the importance of recognizing and addressing gendered narratives in healthcare. As Ahlsen et al. (2014) note, narrative frameworks can provide insight into how cultural expectations of femininity intersect with pain experiences, shaping how women understand and express their pain. Megan and Hailey’s narratives illustrate how these frameworks inform their interactions with healthcare providers and shape their sense of agency in navigating chronic pain. Both participants described how their pain was dismissed, reinforcing their feelings of isolation and requiring them to re-story their experiences to resist dominant narratives of invisibility and invalidation.

While this study focuses on emerging adult women, some narratives—such as dismissal and silencing in healthcare—may resonate across age groups. However, the intersection of chronic pain with the developmental milestones and cultural expectations of emerging adulthood sets this demographic apart. For example, the stigma of being “too young” to experience severe pain appears unique to younger women. Future research could explore how these narratives shift across the lifespan and whether findings from this study can inform broader approaches to chronic pain management for women of all ages.

By making visible the experiences of Megan and Hailey, this study contributes to a growing call for change, urging a move away from singular narratives that stereotype and marginalize women with chronic pain. Recognition of individual pain experiences supports improved diagnosis and reinforces the need for care models tailored to individual needs (de Souza et al., 2017; de Souza Costa et al., 2016). This narrative inquiry demonstrates that the knowledge to be heard, validated, and trusted is vital for individuals navigating chronic pain, offering greater insight into the sociocultural context of healthcare encounters.

### Strengths and Limitations

To our knowledge, this study is the first narrative inquiry to explore the impact of chronic pain in emerging adult women. The intent of this narrative inquiry was not to generalize findings to the broader chronic pain population, but to elicit a deeper understanding of experience. Ethnicity was not considered in the exclusion or inclusion criteria; however, both participants were of White ethnicity. The first author shared common characteristics with the participants, including lived experience of chronic pain and navigating healthcare systems as an emerging adult woman. These shared experiences fostered trust and rapport with participants, creating a space for open and authentic storytelling. For instance, participants expressed feeling understood without needing to provide extensive background explanations of their pain experiences. However, these shared characteristics required careful reflexivity throughout the research process to ensure that the first author’s interpretations were grounded in participants’ narratives rather than shaped by personal assumptions. Reflexive journaling and engagement with response communities were used to critically examine and mitigate potential biases in data collection and analysis.

Narrative inquiry involves small, purposive samples to deeply explore individual experiences. This approach allows for rich, detailed data collection, focusing on the depth and nuance of personal stories rather than broad generalization. However, the limited number of participants in this study may affect the transferability of the findings to broader populations. Furthermore, both participants in this study were university students and shared narratives of being high achievers, which may have influenced their narratives and the way they storied their chronic pain experiences. For example, both Hailey and Megan described how their educational goals shaped their identity and interactions with healthcare providers. While these characteristics were not deliberate selection criteria, they reflect a potential limitation of this study. Future research should explore the experiences of emerging adult women from more diverse educational and socioeconomic backgrounds to provide a broader understanding of how chronic pain intersects with identity formation.

### Future Directions

While the study’s focus on two participants allowed for deep exploration of their narratives, this small sample size may limit generalizability from outside of a narrative inquiry perspective. Additionally, the reliance on online recruitment and community partners for recruitment may have excluded individuals who lacked access to these networks. Future studies could broaden recruitment strategies to capture more diverse perspectives while maintaining the depth required for narrative inquiry.

Additional research is needed to understand the chronic pain experiences of emerging adult women. Longitudinal studies could address existing gaps in knowledge on etiology, incidence, treatment effects, and outcomes of chronic pain in emerging adults (Brown et al., 2021). Studies that examine how tailored interventions can improve quality of life when designed in the context of age and gender are needed. Given that the last Canadian prevalence study that provides chronic pain estimates for emerging adult women was published in 2011 (Schopflocher et al., 2011), current population prevalence studies are necessary to evaluate the present burden of chronic pain in emerging adult women.

This study was intentionally designed to explore the lived experiences of emerging adult women with chronic pain, focusing on how gendered societal narratives shape their experiences. Future research could also explore how gender stereotypes intersect with chronic pain across men and non-binary individuals. This study highlights the need for greater recognition of the gendered and age-specific narratives that influence how chronic pain is experienced and addressed among emerging adult women. By integrating these lived experiences into healthcare practices and research, it is possible to develop more inclusive care models that validate women’s pain and provide individualized support. Understanding the difference between sex (biological differences) and gender (socially constructed roles) is also critical to understanding the unique challenges faced by women with chronic pain. Finally, addressing the underrepresentation of women in medical research and ensuring their voices are heard in healthcare decision-making can help to dismantle narratives that minimize or dismiss their pain experiences. Social change may be small to begin with, but more often than not, it begins with a story (Brass, 2018; Clandinin et al., 2018; Kubota, 2017).

There is a recognized need to explore how pain affects certain populations differently to address the burden of pain within Canada (CPTF, 2019). Given that chronic pain worsens with age (Syx et al., 2017) and adults regulate their emotions differently as they age (Barnes, 2018), understanding the experiences of women with chronic pain in emerging adulthood is crucial to early and successful management of chronic pain.

## Conclusion

In this paper, key findings were presented from a thesis study that explored how living with chronic pain shapes the identity of emerging adult women. The study utilized Clandinin and Connelly’s (2000) narrative inquiry methodology, which views human experience as a valuable source of knowledge and emphasizes the role of stories in understanding identity formation within personal and social contexts.

Narrative accounts of the two emerging adult women, Hailey and Megan, showed dismissal through familial, cultural, and institutional narratives over time that shaped how they storied themselves, leading to decreased self-esteem and silencing of their own needs. Their accounts are stories of being silenced in spaces of health care, and being storied by others as exaggerating, malingering, or their pain purely psychological. Combined with family narratives of pain dismissal centered around their respective mothers, feelings of invalidation influenced personal narratives about disability and self-worth, and Hailey and Megan learned to silence their own pain experiences to avoid being dismissed or disappointed by others.

The stories of those experiencing pain, illness, or disability are often not heard without alteration or fragmentation by researchers (Holloway & Freshwater, 2007) and in this inquiry, the experiences of two emerging adult women with chronic pain were made visible. By increasing awareness and recognizing the unique experiences of emerging adult women with chronic pain, shifts in medical and societal discourses can begin to occur that better reflects and address the needs of this underserved demographic.

## Notes

1. Pseudonyms were used and chosen by each participant.
